# NEUROPSYCHIATRIC SYMPTOMS AND FUNCTIONAL ACTIVITIES AMONG OLDER ADULTS: THE ROLE OF ETHNICITY AND COGNITIVE STATUS

**DOI:** 10.1093/geroni/igz038.1152

**Published:** 2019-11-08

**Authors:** Shanna L Burke, Adrienne Grudzien, Mitra Naseh, Tamara J Cadet

**Affiliations:** 1 Florida International University, Miami, Florida, United States; 2 Simmons University, Boston, Massachusetts, United States

## Abstract

Little is known about the likelihood of future functional deficits based on current neuropsychiatric symptoms (NPSs). This study seeks to examine the impact of NPSs on functional activities (FAs) by cognitive status and ethnicity. A secondary analysis of the National Alzheimer’s Coordinating Center Uniform Data Set was conducted using ordered logistic regression to examine the effect of NPSs (based on the Neuropsychiatric Inventory Questionnaire [NPI-Q]) on FAs (based on Functional Assessment Questionnaire). Participants had a mean age of 74 (SD: 9.88) and were included if normal cognition was assessed at baseline (n= 13,470). Higher rates of NPSs were associated with higher dependency in almost all FAs. Among NPSs, apathy was the best predictor (p<.05) of FAs for participants in different cognitive groups and ethnicity subsamples. The impact of other NPSs varied. Anxiety and apathy were the best predictors of FAs among participants with cognitive impairment (but not MCI). Among those who eventually developed dementia (n= 6,818), delusions, hallucinations, agitation, depression, irritability, and motor disturbance were significantly associated (p<.05) with future deficits in FAs. Among Hispanics (n=1,095), hallucinations, agitation, apathy, and motor disturbance were significantly associated with dependency in FAs, while for non-Hispanics, all NPSs were associated with dependency in FAs, except elation and nighttime disturbance. Findings suggest as the severity of the NPSs increases, older adults experience higher levels of dependency in FAs. The nature and extent of NPSs’ impact on FAs varied based on cognitive status and ethnicity, suggesting the importance of considering these factors in service provision.

